# Postoperative cataract care: the Aravind perspective

**Published:** 2016

**Authors:** Aravind Haripriya, Zervin R Baam, RD Ravindran

**Affiliations:** Chief: Cataract services, Aravind Eye Hospital, Madurai, India.; Fellow: Cataract Services, Aravind Eye Hospital, Madurai, India.; Chairman: Aravind Eye Care System, Madurai, India.

**Figure:**
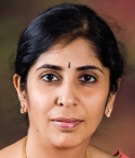
Aravind Haripriya

**Figure:**
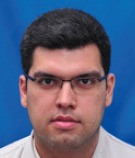
Zervin R Baam

**Figure:**
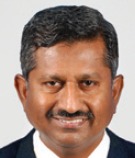
RD Ravindran

“Your operation has been a success.”

It is such a relief for a patient to hear these words from the operating ophthalmologist. All is well that ends well. However, it is essential for us as eye care professionals to make the patient aware that the end of a successful cataract operation is not the end of her or his treatment for cataract. Good postoperative care is as important as the care taken by the surgeon before and during surgery. At Aravind Eye Hospitals, we make a point of stressing good postoperative care, especially since excellent surgery can have a poor outcome if the postoperative care is not adequate.

At Aravind many of our patients are operated on as day cases and others are admitted, particularly patients identified with cataract during outreach activities. For all cataract operations the procedure ends with the administration of intracameral antibiotics (for endophthalmitis prophylaxis). We use intracameral moxifloxacin for this, which has reduced postoperative endophthalmitis from 8 per 10,000 operations to 2 per 10,000.^1^ Following this, a drop of 5% povidone iodine is instilled into the conjunctival sac and the eye is patched.

**Figure:**
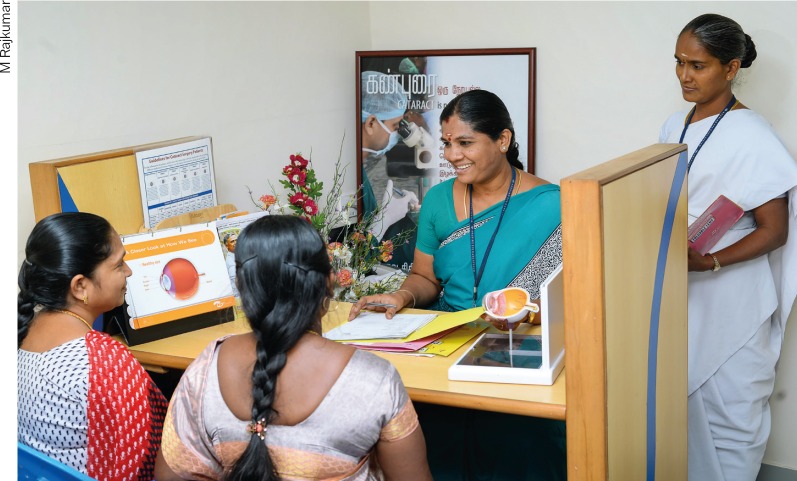
A patient counsellor explains cataract surgery and the various types of implants (intraocular lenses [IOL]) to the patient and relatives. INDIA

Patients are taken to a recovery room, where they are counselled regarding care to betaken during the postoperative period. The correct method of instilling drops is shown to them during counselling. Patients are instructed to clean the lid margins and adjoining area with surgical cotton (given to them in their postoperative medicine kit) and advised not let any fluid or foreign body enter the eye. Dark glasses (sunglasses) are recommended to be worn outdoors for protection and to reduce glare.

**‘Good postoperative care is as important as the care taken by the surgeon before and during surgery’**

Day surgery patients are sent home wearing an eye patch and eye shield and instructed to do the following on the day of surgery:

- Remove the eye patch and eye shield 2–3 hours after reaching home.
- A total of 6 hours after surgery, instil antibiotic drops every hour (5 to 6 times in total) and topical steroid drops every two hours (3 times in total). From the next day, the steroids are increased to 6 times a day and then slowly tapered.
- Wear the sterile eye patch (given to patients on discharge) on the first night after surgery.

For inpatients, the same regime is followed except that the eye drops are administered by nurses.

Alongside the use of intracameral antibiotics and topical povidone iodine at the time of surgery, topical antibiotic drops in the immediate postoperative period help to prevent infection caused by contamination of the anterior chamber during surgery or in the immediate postoperative period.

If the posterior capsule has been ruptured during surgery, patients are started on a course of systemic antibiotics (ofloxacin 200 mg twice daily) on the day of surgery for a period of 3 days, as additional prophylaxis against endophthalmitis.

All patients are examined on the first day after surgery in order to rule out any early postoperative complications, to diagnose any fundus pathology which was not possible pre-operatively due to media haze, and to assess the immediate postoperative visual outcome.

Examination includes measurement of visual acuity with and without a pinhole, slit lamp examination and fundus examination. The patients' pupils are dilated prior to examination. Particular attention is paid to the cataract wound (to check whether the wound has opposed well), the clarity of the cornea, the anterior chamber depth, cellular reaction and the location and centration of the intraocular lens. A second operation is planned if there is wound leak, significant retained lens matter or a decentred intraocular lens (IOL). Fundus examination is undertaken using a +90D lens to rule out glaucoma or retinal pathology.

In the event of posterior capsular rupture (PCR), more detailed examination is required. The presence of vitreous in the anterior chamber is checked, to see whether it is touching the cornea (causing pupillary block) or whether strands of vitreous are incorporated into the wound. In the case of the latter, Nd:YAG vitreolysis is planned, as well as surgical anterior vitrectomy in case of vitreous in the anterior chamber. In the event of a PCR, detailed examination of the vitreous cavity is also required to rule out dislocated nuclear or cortical material.

On the first postoperative day (day 1), patients are counselled again; the need for follow up after 30 days is emphasised and the postoperative medication regimen is explained. Patients are also advised to see an ophthalmologist immediately if they have sudden pain, redness or decreased vision.

**Figure 1.**
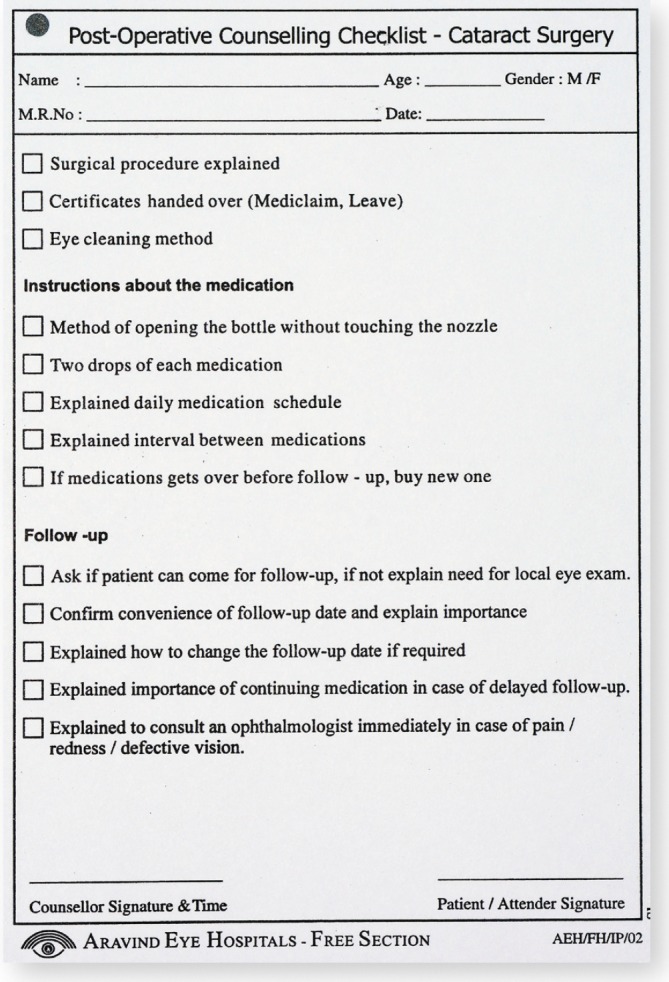
A counselling checklist helps to ensure good communication with the patient

At Aravind, great importance is given to counselling: specially-trained counsellors are given the job of adequately advising patients and answering any questions they may have. At the end of counselling, a checklist is used to ensure all relevant aspects are communicated to the patient (Figure 1). Patients are also told when they can resume various activities.

Most patients who were admitted are discharged on the first postoperative day; however those coming as a result of our outreach activities are discharged on the second or third day depending on the distance to their village. A detailed discharge summary is given which includes the details of the postoperative medications, i.e. topical steroid and antibiotic eye drops.

Steroids are used 6 times per day for the first week and tapered each week over 6 weeks. Topical antibiotics are used 3 times daily for 2 weeks postoperatively. Topical non-steroidal anti-inflammatory drugs (NSAIDs), such as nepafenac or ketorolac, are generally started routinely at the 1 month follow-up visit to reduce the incidence of cystoid macularoedema (CME) and to prevent rebound inflammation, which may occur after the steroids are tapered and stopped. In high risk cases such as PCR with vitreous loss, or if there was CME following surgery to the first eye, NSAID drops are started immediately after surgery.

At the first month follow-up visit, patients undergo refraction and spectacles are prescribed based on their needs. Their intraocular I pressure is checked to rule out raised intraocular pressure following the use of topical steroids. The eye is examined comprehensively for any sequelae following surgery and the other eye is assessed in order to plan the timing of a cataract operation on the second eye, if needed. Some patients may be called back sooner than one month if there were any surgical or postoperative complications.

Patients are advised to undergo an annual follow-up examination after surgery in both eyes.

Whereas the surgeon and eye team have a greater role to play in the surgical care, postoperative care involves sharing responsibility between the eye team and the patient. It is crucial that the patient is educated regarding the importance of compliance with treatment and follow-up in order to ensure an excellent outcome.
